# Extirpation of the Right Parotid Gland

**Published:** 1868-11

**Authors:** Wm. C. Crcoks


### ARTICLE X.

### *Extirpation of the Right Parotid Gland.*

By Wm. C. Crcoks, M. D.

The following case, a large, robust, plethoric, and corpulent Irish woman, æt 45, came under my care August 25th, 1866. In June, 1866, during an altercation she had with other parties, she received a blow immediately below the right ear, and soon after this noticed a swelling of that side of the face. It increased slowly and without much pain for nearly two months, and was thought to be a fatty tumor, or perhaps an abscess. The latter diagnosis being thought the most probable, appropriate applications were made to hasten suppuration. The tumor yielded readily to this treatment, and when sufficiently soft and fluctuating was punctured; when a large quantity of ichor was discharged of an ulcerous nature. About the beginning of August, other openings appeared anterior and posterior to the first, from which a thin fœtid fluid issued; and, when I first saw her, there was not only the opening first made by the lancet, which had now sloughed to a considerable extent, but around it were numerous smaller ones. From all these apertures pus was freely discharged, and accompanied by large sloughs which not unfrequently required for their extrication the use of the forceps.

The submaxillary gland, and the lymphatics of the right side of the neck were more or less involved; and, down the neck till within one and a half inches of the sternal origin of the sterno-cleido-mastoid muscle, and anteriorly on the face the masseter muscle, and posteriorly on the neck the sterno-cleido-mastoid were partially bare, and could be seen, owing to the undermining of the parts by the sloughing. A large pouch was formed by the skin and subjacent tissue of the neck, which served as a receptacle for the gravitating fluid. The large blood vessels of the neck, and some of their larger branches in the parotid region, could be seen by dexterously separating the sloughing parts. From the latter a troublesome hemorrhage more than once occurred.

Such a case as this needs little comment. But one thing could be done, and that was to remove the gland entire, and arrest the sloughing as quickly as possible. For the important blood vessels of the neck had already become endangered, and their destruction inevitable if a change was not effected and that without delay. The operation was as follows: One crescentic incison was made, from the zygoma to a point two inches behind the ear on the os occipitis, the sloughing having provided amply for the incision on the neck. On detaching the integument in the form of a double flap, the gland was fully exposed, and as expected, found in a condition favoring easy detachment. Commencing behind the gland, and by working forward it was gradually freed from its posterior and inferior attachments. The external carotid artery was now secured, and the upper and anterior attachments divided, and the gland removed from its deep adhesions. These were by no means as close as in the healthy condition, for a large portion of the gland was was removed in the form of small shreds and sloughs, and such adhesions as were yielded readily. During the operation the temporal, transverse-facial and internal maxillary arteries were cut and ligated. The submaxillary gland being involved in the disease, along with a number of thə lym. phatics in the vicinity, was also removed. A slight hemorrhage which yielded readily to styptics occured. The wound was then filled lightly with lint well saturated in a strong solution of permanganate of potassa and liquor sodæ chlorinatæ. I will here append the recipe used on that occasion:

R. Potass. Per-mang , ʒ ij.

Liq. Sodæ Chlor., f. ℥ ij. M.

S. Tablespoonful to be added to Oss. water.

With this dilution the edges of the flaps were well washed, and then closed by adhesive strips, and covered with a compress, and bandage. The next day this dressing was removed and a stimulating poultice applied, having the wound well washed twice daily with the above formula ; but of a greater pilution, using only a dessert-spoonful to the pint of water. This was continued three days, when I applied a solution of nitrate of silver, 20 grains to the ounce, and ordered to follow it the ceratum resinæ. Under this the patient improved rapidly, and in seven weeks was perfectly well. The patient removed from the city three months after the operation, where I know not, and I have not heard from or of her since. Therefore I am unable to ascertain whether or not the disease has again returned.

It is almost superflous to observe that during all this time of sloughing, suppuration and reparation, the patient had been well managed, and amply supported with food, and wine, and medicine.

At first it was very difficult to arrive at any well grounded opinion as to the true nature of this disease on account of inability to trace any hereditary transmission of the cancerous diathesis. The patient assured me that she was well acquainted with the statistics of her family ancestry, and, that they in general enjoyed both health and longevity, showing at no time any evidence of the cachexia. I questioned her closely, but only found her, if my conjectured diagnosis proved correct, to be unlike her ancestors; which after more accurate and minute examination was proven beyond a doubt.

The cachexia may and undoubtedly does sometimes become subjected to a partial or entire eradication by the production of tumors, whose character are intermediate or transitional between cancerous and simple growths; and may, in this process of unloading the system of that morbid condition of the blood escape the cancerous character, but still be transmissable to posterity. Therefore it can readily be conceived how the parent tainted with the cachexia, though not manifestly so, (on account of the morbid material in the blood being somewhat deficient, and with this no tissue favoring its localization,) may transmit the disease to their offspring, who may offer a better soil for the seed to germinate and mature.

I believe that in a great number (if not all) of the cases where hereditary transmission is so difficult and seemingly impossible to trace, that by casting a scrutinizing and searching inquiry back into ancestry the germ will be discovered; and not unfrequently in this way, viz., that at some time or other there existed in some member of the family growths which, though receiving the ordinary appellation of tumors, would have been found if subjected to an examination under the microscope to contain cancer cells; but so altered, that, as I said before, the cancerous disposition was gradually fading out. Suppose a case of scirrhous, or medullary, or any of the many different varieties of cancer should come under observation wherein no hereditary nature could be traced, and that in that individual case there was no such predisposition traceable. There could be left only two other probable sources, those of inoculation and contagion: but concerning these the presumed facts are, at present, very few and uncertain.

Convinced that in the case under observation the tumor was unquestionably a cancerous one, I resolved if possible to trace out the hereditary transmission, or at least satisfy myself better in regard to it. When talking with the patient on one occasion, she told me that she had had recalled to her memory by a friend, when conversing on this subject, what I considered an all important point, i. e., the appearance of hard fibroid tumors (so called) more than once on her grand-parent. Now this may be said to be mere conjecture or wild speculation; but may we not deduce from it that the cancerous substance in the blood whatever it may be, and whencesoever derived, is a result of long continued elaboration? The feasibility of this question at least will be admitted.

But now let it be observed, this tendency to cancerous disease is often and most generally derived from a parent, who is not yet manifestly cancerous; and the tendency which exists in the parent may even succeed that in the child in developing, or it may become evident in the parent, and (though the tendency exist,) never become effective in the child, and vice versa. Thus it is handed down from grand-parent to grand-child, while in the interposed generation it remains occult.

It is almost superfluous for me here to observe the well known fact of transmutation during transmission of cancer from parent to child; and while this is supported by actual observations in practice, it is at least possible that I may be justly attributing the cause in this case to hereditary disposition. If the tumors which appeared on the grand-parent were cancerous, (and I believe they were,) they without a doubt were of the scirrhous or hard variety; while that in the patient was of the medullary, going to add another testimony in support of the now well proven fact, i. e., that in hereditary transmission the cancer material may be modified so that the form of the disease in the offspring may be different from that in the parent. The change from scirrhous to medullary cancer, and vice versa, is not an unfrequent tale.—*Med. and Surg. Reporter.*

### MONTHLY SUMMARY.

